# Development of immunoassays for detecting oxyfluorfen residue in agricultural and environmental samples†

**DOI:** 10.1039/c7ra12445g

**Published:** 2018-01-30

**Authors:** Enze Sheng, Mei Du, Jiachuan Yang, Xiude Hua, Minghua Wang

**Affiliations:** Department of Pesticide Science, College of Plant Protection, Nanjing Agricultural University Nanjing 210095 P. R. China wangmha@njau.edu.cn +86 25 84395479 +86 25 84395479; State & Local Joint Engineering Research Center of Green Pesticide Invention and Application Nanjing 210095 P. R. China

## Abstract

An enzyme-linked immunosorbent assay (ELISA) and chemiluminescent immunoassays (CLEIA) were developed to detect oxyfluorfen in agricultural and environmental samples. The hapten of oxyfluorfen was synthesized and conjugated with bovine serum albumin (BSA) and ovalbumin (OVA) to produce immunogen and coating antigen. One cell line (1A7D6F5) that stably secretes anti-oxyfluorfen monoclonal antibody (mAb) is obtained by cell fusion. Under optimal conditions, the half-maximal inhibition concentration (IC_50_) and the limit of detection (LOD, IC_20_) of ELISA are 0.065 mg L^−1^ and 0.0048 mg L^−1^, while those of CLEIA are 0.021 mg L^−1^ and 0.0016 mg L^−1^, respectively. The immunoassays show no obvious cross-reactivities with the analogues of oxyfluorfen except for benzofluorfen and bifenox. The recoveries of oxyfluorfen in the spiked samples of soil, grape, peach, apple and pear are in the range of 74.1–107.2% with a RSD of 2.7–9.7% for ELISA, and 77.2–106.4% with a RSD of 2.4–7.9% for CLEIA. The results of immunoassays for the authentic samples are significantly correlated with those detected by gas chromatography (GC).

## Abstract 2

An enzyme-linked immunosorbent assay (ELISA) and chemiluminescent immunoassays (CLEIA) were developed to detect oxyfluorfen in agricultural and environmental samples.

## Graphical abstract

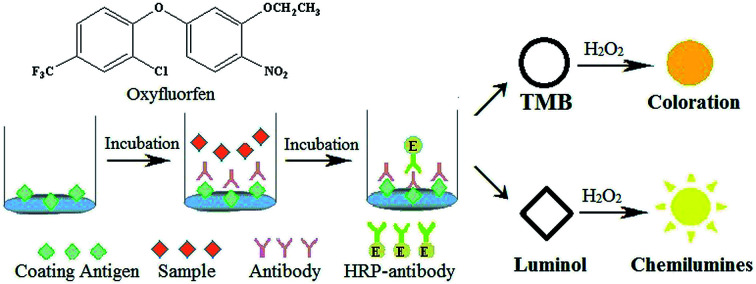

## 1. Introduction

Oxyfluorfen (2-chloro-α,α,α-trifluoro-*p*-tolyl-3-ethoxy-4-nitrophenyl ether) is a kind of fluorinated diphenyl ether herbicide that was developed by Rohm and Hass in 1975. It is a commonly used pre-or-post emergent herbicide for broadleaved and grassy weeds in a variety of field crops.^1^ Oxyfluorfen is highly toxic to aquatic invertebrates, wildlife and fish, and its biodegradation is known to be very poor. What is more, plants cannot metabolize oxyfluorfen and it can be only slowly assimilated by microorganisms.^2^ Due to the characteristics of oxyfluorfen, government agencies around the world, such as the United States Food and Drug Administration (FDA) and Health Canada have revised the maximum residue limit of oxyfluorfen several times since 2014. So, it is necessary to establish rapid, highly sensitive, economical and friendly methods for the detection of residual oxyfluorfen.

Currently, several instrument-based methods, such as gas chromatograph (GC)^3,4^ and liquid chromatography (LC)^5,6^ are commonly used to detect oxyfluorfen in environmental and agricultural samples. GC coupled to mass spectrometry (GC-MS)^7–10^ has been the most popular method to detect the oxyfluorfen residue in recent years. These methods depend on expensive instrumentations and the use of large organic solvent. Immunoassays have the advantages of rapid, simple, sensitive, high throughput, economical and friendly to environment.^11–16^ As a frequently-used immunoassay, enzyme-linked immunosorbent assay (ELISA) has been proved to be a rapid and sensitive method for quantitative and qualitative detection of compounds.^17–19^ The chemiluminescent enzyme immunoassay (CLEIA) also has gained attention in the research of clinical diagnosis and analytical test because of its higher sensitivity and wider dynamic range of linearity compared with colorimetric detection.^20^ These advantages demonstrate CLEIA is suitable for quantitative detection of analyte with high sensitivity.

In this paper, a hapten of oxyfluorfen was designed and synthesized to prepare anti-oxyfluorfen monoclonal antibody (mAb). An ELISA and a CLEIA were developed to detect the residual oxyfluorfen in agricultural and environmental samples. The immunoassays were evaluated by cross-reactivity (CR) and spiked recovery after optimization, and verified with GC through analysis of authentic samples.

## 2. Materials and methods

### 2.1. Chemicals and instruments

Oxyfluorfen (95.3%) and the pesticide standards used for cross-reactivity studies were supplied by Jiangsu Academy of Agricultural Sciences (Jiangsu, China). Bovine serum albumin (BSA), ovalbumin (OVA) were purchased from Sigma Chemical Co. (St. Louis, USA). Dulbecco's modified eagle medium (DMEM), hypoxanthine aminopterin thymidine medium (HAT medium) and hypoxanthine thymidine supplement medium (HT medium) were bought from Gibco Co. (California, USA). Goat anti-mouse immunoglobulin–horse radish peroxidase (GAM–HRP) conjugate was purchased from Bister Biological Technology Co., Ltd (Hubei, China). 3,3′,5,5′-Tetramethyl benzidine (TMB), luminol, H_2_O_2_, *p*-iodophenol, polyoxyethylene sorbitan monolaurate (Tween-20), tris(hydroxymethyl)aminoethane (Tris) and other chemical reagents were purchased from Aladdin (Shanghai, China). The BALB/c mice were obtained from the Center of Comparative Medicine of Yangzhou University (Yangzhou, China). All animal experiments in this study was performed in strict accordance with the guidelines for the care and use of laboratory animals (license number SYXK (SU) 2017-0044) and was approved by the Department of Science and Technology of Jiangsu Province (Jiangsu, China).

Electrospray ionization-mass spectrometry (ESI-MS) data were obtained with a LC-MS^QDECA^ (Finnigan, USA). Nuclear magnetic resonance (NMR) spectra were recorded on a DRX 500 spectrometer (Bruker, Germany). Ultraviolet-visible (UV/vis) spectra were obtained on a UV-2550 spectrophotometer (Shimadzu, Japan). Microtiter plates were 96-well transparent microplates (Jet Biofil, China) and 96-well white microplates (Corning, USA), respectively. The microplates were washed using an Immuno Wash 12 (Thermo, USA). The absorbance and chemiluminescence emission were detected using a SpectraMax M5 (Molecular Devices, USA). The ELISA and CLEIA were validated with an Aglient 7890A GC (Aglient, USA).

### 2.2. Hapten synthesis and verification

**Fig. 1:**
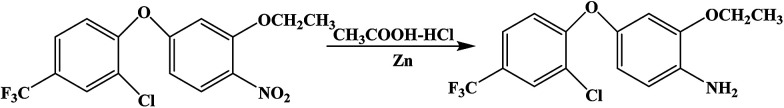
Synthetic route to oxyfluorfen hapten.

The hapten synthetic route is shown in Fig. 1. 4 g oxyfluorfen was dissolved in 40 mL ether, the solution was washed 3 times by 20 mL Na_2_CO_3_ (0.1 mol L^−1^) to remove the impurities. Then, 8 g Zn powder and 25 mL acetic acid : hydrochloric acid solution (v/v, 9 : 1) were added into the solution, and stirred for 1 h at 45 °C. The ether layer was washed by water (2 × 30 mL) and dried over by anhydrous sodium sulfate. After concentration, the brownish yellow oil was adjusted to pH 2 using 1 mol L^−1^ HCl and extracted with *n*-hexane (3 × 30 mL). The aqueous phase was adjusted to pH 10 by 2.5 mol L^−1^ NaOH and extracted with dichloromethane (3 × 30 mL). The organic phase was evaporated to get brown oil, and purified on a silica gel plate using methanol–trichloromethane (2 : 3, v/v). The second fraction was collected and characterized by ESI-MS and NMR: ESI-MS, *m*/*z*, 332 [M + H]^+^ and 354 [M + Na]^+^; ^1^H NMR (400 MHz, CDCl_3_) *δ*: 7.69 (s, 1H, ArH), 7.36 (d, 1H, ArH), 6.82 (d, 1H, ArH), 6.71 (d, 1H, ArH), 6.56 (d, 1H, ArH), 6.51 (dd, 1H, ArH), 4.01 (q, 2H, OCH_2_), 3.83 (s, 2H, NH_2_), 1.43 (t, 3H, CH_3_).

### 2.3. Preparation of hapten–protein conjugates

The hapten was conjugated with BSA and OVA by the glutaraldehyde cross-linking method to produce immunogen and coating antigen.^21,22^ 10 mg hapten were dissolved into 0.3 mL methanol, and then added 0.7 mL water (solution I); 5 mg BSA were dissolved into 0.35 mL phosphate-buffered saline (PBS, 0.01 mol L^−1^, pH 7.2) (solution II); 100 μL glutaraldehyde were added into 5 mL water (solution III). Then the solution I and III were gradually added dropwise into the solution II at room temperature with stirring. After the dripping, solution was stirred for 8 h. The conjugates were dialyzed in PBS over 72 h at 4 °C and stored at −20 °C. The coating antigen was prepared by the same method. The conjugates were verified by UV/vis spectroscopy, and the mole absorbance at 280 nm was used to estimate their hapten densities (the number of hapten per molecule of protein) by the equation: hapten density = (*ε*_conjugate_ − *ε*_protein_)/*ε*_hapten_.

### 2.4. Immunization and mAb preparation

Five 6 week-old female BALB/c mice were used to perform immunization experiments by intraperitoneal injection of immunogen. The timetable was based on the method described by Kishiro.^23^ The first injection for each mouse was 100 μg immunogen, which was diluted in physiological saline and emulsified with an equal volume of Freund's complete adjuvant. Three weeks later, the booster shoots were given 4 times at intervals of 2 weeks with an incomplete Freund's adjuvant. Since the 3rd immunization, 1 week after the each immunization, blood samples were drawn from the tail veins to check the titer of the antibodies. 1 week after the last immunization, another 200 μg of immunogen in PBS were injected. 3 days after the injection, cell fusion was performed according to Nowinski.^24^ Mouse spleen lymphocytes were fused with SP2/0 myeloma cells at a 5 : 1 ratio. The fused cells were cultured in HAT medium in an atmosphere of 37 °C, 5% CO_2_. Every 3 days, half of the medium was replaced with fresh HAT. After 14 days of cell fusion, HT medium was used in place of HAT. The culture supernatants in 96 well plates were screened by ELISA, and the positive wells were subcloned to obtain hybridoma cell line by restriction dilution. The ascites were purified using caprylic acid–ammonium sulphate, and then stored at −20 °C after freeze-drying.^25^

### 2.5. Immunoassay procedures

The ELISA was performed on 96-well transparent microplates. The coating antigen was diluted with carbonate-buffered saline (CBS, 0.05 mol L^−1^, pH 9.6), and added to the microplates (100 μL per well) for incubation at 4 °C overnight. The plates were washed 5 times with PBS containing 0.05% Tween-20 (PBST), and then 3% skim milk in PBS (200 μL per well) was used to block the plates for 1 h at 37 °C. After another washing step, the mAb (50 μL per well) and sample or standard solution (50 μL per well) were added and incubated for 1 h at 37 °C. Following a wash step, the diluted GAM/HRP (100 μL per well, 1 : 20 000 in PBS) was added and incubation at 37 °C for 1 h. After washing, the amount of bound enzyme was measured by adding 100 μL per well peroxidase substrate (1 L citrate buffer (pH 5.0) contained 0.4 mmol TMB and 3 mmol H_2_O_2_). The absorbance at 450 nm was determined after the reaction (37 °C, 15 min) was stopped by adding 50 μL per well of 2 M H_2_SO_4_.

For CLEIA, 96-well white microplates were used, and the procedure was similar to ELSIA. The amount of bound enzyme was measured by adding 150 μL per well of freshly enhanced chemiluminescence solution (1 mmol L^−1^ luminol, 0.025 mmol L^−1^*p*-iodophenol and 1.5 mmol L^−1^ H_2_O_2_ in Tris–HCl buffer (0.1 mol L^−1^, pH 8.5)). After 10 min reaction at 37 °C in the dark, the chemiluminescence intensity (relative light units, RLU) was detected with 1 s integration time at 435 nm.

### 2.6. Immunoassay optimization

The two-dimensional checkerboard method was used to confirm the optimal concentrations of coating antigen and antibody. In order to improve the sensitive of immunoassay methods, the experimental parameters including the organic solvent (methanol, 0% to 50%, v/v), ionic strength (Na^+^, 0.1 to 0.6 mol L^−1^) and pH value (pH 4.5 to 9.5) were investigated. The evaluation of ELISA or CLEIA was based on the IC_50_ and the maximum of absorbance (*A*_max_) or RLU (RLU_max_). The combination of lowest IC_50_ and highest *A*_max_/IC_50_ or RLU_max_/IC_50_ was the most desirable.

### 2.7. Cross-reactivity

CR was studied using the standard solution of the oxyfluorfen and its analogues. The CR values were calculated as follows: CR% = (IC_50_ of oxyfluorfen/IC_50_ of analogue) × 100.

### 2.8. Analysis of spiked samples

Soil (got from a farm in Nanjing, China), grape, peach, apple and pear (bought from a market in Nanjing, China) were employed to study spiked recoveries. The samples were verified without oxyfluorfen by GC, and spiked with oxyfluorfen at 0.05, 0.1, 0.5 mg kg^−1^. The spiked samples stored overnight at room temperature. The samples were extracted twice by sonication for 10 min in 10 mL of methanol and centrifugation at 4000 rpm for 10 min. The supernatants were diluted appropriate times by PBS and analyzed by ELISA and CLIEA. The experiment of each sample was conducted in triplicate. The recoveries and relative standard deviations (RSD) were calculated.

### 2.9. GC analysis and validation

Soil samples were collected from farms where oxyfluorfen 24% EC had been used in Nanjing, China. The extraction and analysis of immunoassays were the same with the spiked samples. For GC, 20 g soil samples were vigorously shaken with 10 mL water and 60 mL acetonitrile for 1 h. After the organic phase was dehydrated and concentrated, the samples were diluted with 2 mL of acetone and further confirmed by GC-ECD.^3,4^ The GC column was a DB-1701 fused silica capillary column (30 m × 250 μm × 0.25 μm), and nitrogen was used as the carrier gas. The column temperature was initially held at 120 °C for 1 min, then raised to 270 °C at 20 °C min^−1^ and held for 3 min. The measured results were compared with the results from the ELISA and CLEIA.

## 3. Results and discussion

### 3.1. Conjugation identification and mAb

With the identification by ESI-MS and NMR (Fig. S1 and S2†), the nitro group of oxyfluorfen is successfully reduced to amino. The UV/vis spectra show qualitative differences between the hapten, carrier protein, and conjugates (Fig. S3†), which proved that the hapten with carrier protein were successfully conjugated. The molar ratios (hapten : protein) of immunogen and coating antigen are 15 : 1 and 8 : 1, respectively. After the immunization, one cell line (1A7D6F5) that stably secretes anti-oxyfluorfen mAb was obtained by cell fusion, and the type of mAb is IgM.

### 3.2. Optimization of immunoassays

The optimal concentrations of the coating antigen and antibody for ELISA are 4 μg mL^−1^ and 1 μg mL^−1^, while CLEIA are 4 μg mL^−1^ and 2 μg mL^−1^, respectively. Methanol has the least effect on the antibody–antigen reaction in immunoassays, so it was selected to improve solubility of analysis. As shown in Table S1,† the optimum parameters of ELISA are 20% methanol, 0.5 mol L^−1^ Na^+^ and pH 6.5, while, 10% methanol, 0.5 mol L^−1^ Na^+^ and pH 6.5 are chosen as optimum for the CLEIA.

### 3.3. Sensitivities

**Fig. 2:**
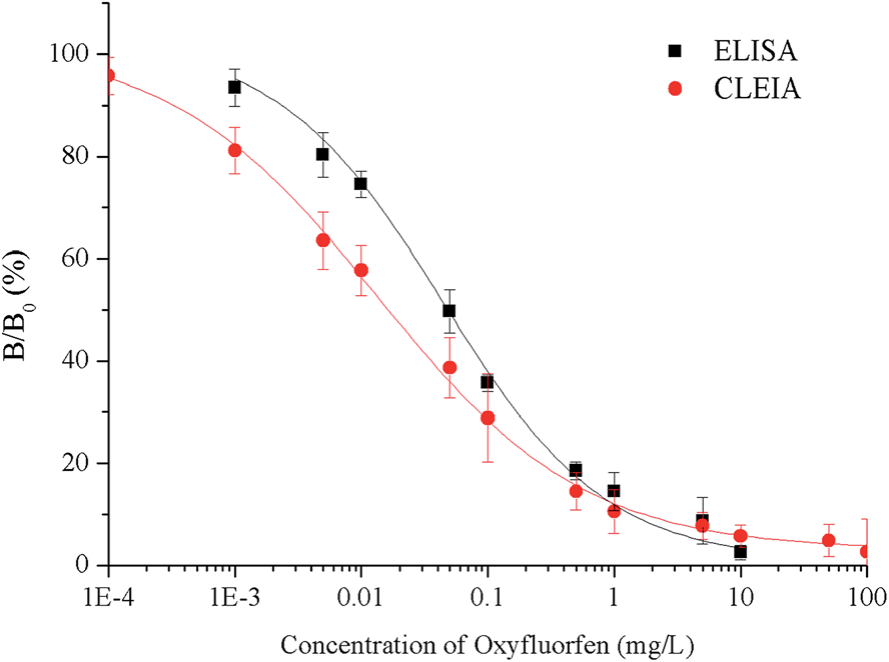
Standard curves for oxyfluorfen by the ELISA and CLEIA.

Under the optimum conditions, the calibration curves are constructed using the relationship between the percent binding (% *B*/*B*_0_) and the concentration of oxyfluorfen (Fig. 2). The ELISA has an IC_50_ of 0.065 mg L^−1^, a LOD of 0.0048 mg L^−1^ and a linear range (IC_20_–IC_80_) of 0.0048–0.63 mg L^−1^. The CLEIA shows higher sensitivity than ELISA with the IC_50_ of 0.021 mg L^−1^, the LOD of 0.0016 mg L^−1^, and the linear range of 0.0016 mg L^−1^ to 0.28 mg L^−1^.

Compared to the maximum residue limit (MRL) of oxyfluorfen in USA (0.2 mg kg^−1^),^9^ the sensitivity of the immunoassays can well meet the requirements. According to the reported articles,^5–8^ the LOD values of HPLC and GC were 0.007 and 0.05 mg L^−1^ respectively, the immunoassays are more sensitive than instrument-based detection methods.

### 3.4. Specificities

The CRs for the analogues of oxyfluorfen were tested (Table 1). The immunoassays show negligible CR with most of analogues (CR% ≤ 2.2%) except for benzofluorfen (16.7% in ELISA and 13.1% in CLEIA) and bifenox (13.5% in ELISA and 9.5% in CLEIA). Therefore, the developed ELISA and CLEIA can specifically detect oxyfluorfen in environmental and food samples.

**Table 1:** Cross-reactivity of a set of analogues structurally related to oxyfluorfen

Compound	Structure	ELISA		CLEIA	
		IC_50_ (mg L^−1^)	CR (%)	IC_50_ (mg L^−1^)	CR (%)
Oxyfluorfen	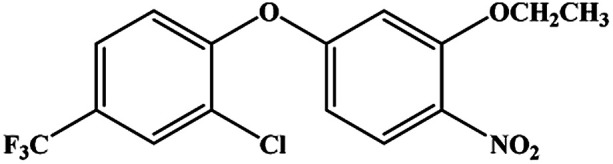	0.065	100	0.021	100
Benzofluorfen	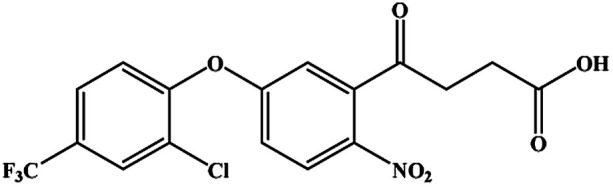	0.39	16.7	0.16	13.1
Bifenox	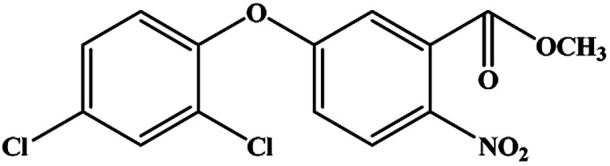	0.48	13.5	0.20	9.5
Fomesafen	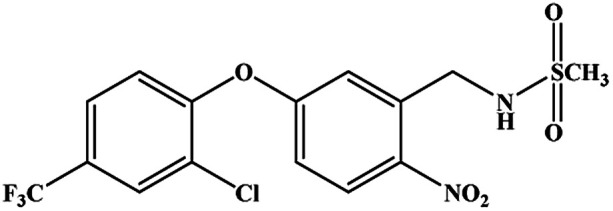	3.02	2.2	2.01	1.0
Acifluorfen sodium	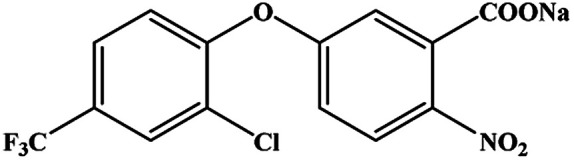	>100	<0.1	>100	<0.1
Lactofen	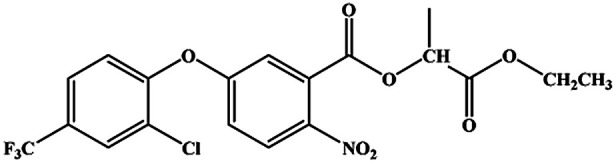	>100	<0.1	>100	<0.1
Nitrofen	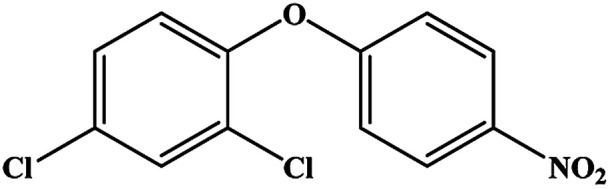	>100	<0.1	>100	<0.1

### 3.5. Matrix effects

The extracts were diluted (5, 10 and 20-fold) with PBS containing methanol (20% for ELISA and 10% for CLEIA). The matrix effects were determined by comparing the standard curves of oxyfluorfen prepared in matrix extract and those standards curves prepared in PBS solution of matrix free. The results of matrix effects show that different sample matrices have variable effects on the sensitivity of the immunoassays, while the matrix effects for two methods are similar. When extracts were diluted 5 times, the matrix effects of grape, peach, apple and pear samples are almost removed. Soil sample matrix shows the greatest impact, 20-fold dilution was selected to remove the effect (Fig. S4 and S5†).

### 3.6. Analysis of spiked samples

The recoveries of the spiked samples were shown in Table 2. The recoveries of ELISA and CLEIA are 74.1–107.2% with relative standard deviations (RSDs) of 2.7–9.7% and 77.2–106.4% with RSDs of 2.4–7.9%, respectively. The accuracy and precision of the immunoassays meet the requirement of quantitative detection according to the guideline on pesticide residue trials of China (NY/T 788-2004). These results indicate that the ELISA and CLEIA are accurate and reliable to determine oxyfluorfen in agricultural and environmental samples.

**Table 2:** Recoveries of oxyfluorfen in spiked samples

Sample	Spiked concentration (mg kg^−1^)	ELISA		CLEIA	
		Mean recovery ± SD (%)	RSDs (%)	Mean recovery ± SD (%)	RSDs (%)
Soil	0.05	—	—	82.6 ± 4.3	5.2
	0.1	107.2 ± 6.4	5.9	77.2 ± 6.1	7.9
	0.5	104.4 ± 4.3	4.1	90.8 ± 2.2	2.4
Grape	0.05	89.1 ± 3.2	3.5	104.5 ± 4.6	4.4
	0.1	93.0 ± 6.4	6.8	105.9 ± 3.9	3.7
	0.5	101.2 ± 3.4	3.3	99.3 ± 7.8	7.8
Peach	0.05	103.7 ± 2.8	2.7	104.3 ± 2.4	2.4
	0.1	90.1 ± 4.6	5.1	106.4 ± 5.8	5.5
	0.5	99.6 ± 4.9	4.9	103.1 ± 7.8	7.6
Apple	0.05	81.4 ± 4.5	5.5	96.4 ± 6.9	7.2
	0.1	89.3 ± 3.2	3.5	95.2 ± 3.1	3.3
	0.5	89.7 ± 2.8	3.1	100.3 ± 6.3	6.3
Pear	0.05	87.0 ± 2.9	3.3	79.3 ± 4.6	5.8
	0.1	89.7 ± 2.8	3.1	83.4 ± 5.8	6.9
	0.5	74.1 ± 7.2	9.7	88.7 ± 6.1	6.9

### 3.7. Analysis of authentic samples

**Fig. 3:**
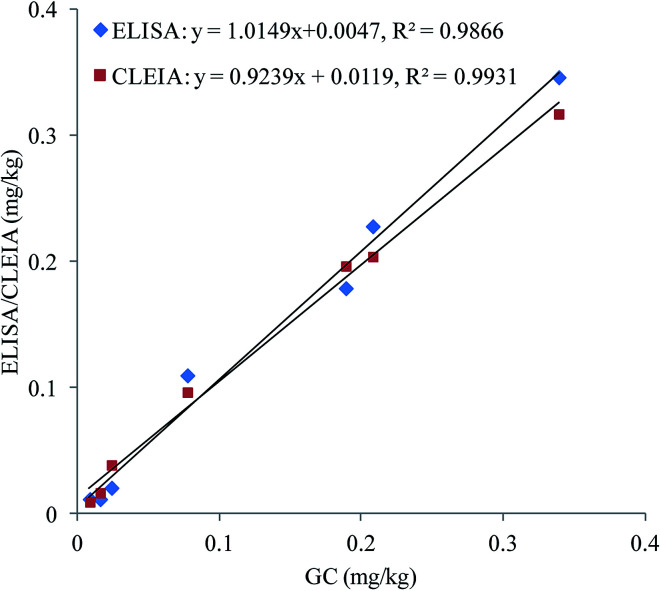
Correlations between the immunoassays and GC for the authentic soil samples.

To confirm the accuracy and precision of the immunoassays, the authentic soil samples were simultaneously analysed by ELISA, CLEIA and GC. As shown in Fig. 3, good correlations are obtained between ELISA and GC (*y* = 1.0149*x* + 0.0047, *R*^2^ = 0.9866) and CLEIA and GC (*y* = 0.9239*x* + 0.0119, *R*^2^ = 0.9931). These results prove that the immunoassays are reliable for quantitative detection of oxyfluorfen in authentic samples.

## 4. Conclusions

In this study, an ELISA and a CLEIA were established to detect the oxyfluorfen in agricultural and environmental samples. The CLEIA provide a lower IC_50_ and LOD (0.021 and 0.0016 mg L^−1^) compared with ELISA (0.065 and 0.0048 mg L^−1^). The specificity and accuracy of these two methods are satisfactory. The results of the authentic samples analysis shown good agreement with GC. All of these results indicate that the two methods can detect oxyfluorfen in agricultural and environmental samples. Based on the same antibody and HRP-conjugate, the luminol/peroxide/enhancer system for HRP provides the possibility of increasing the sensitivity of CLEIA. According to the higher sensitivity, the established CLEIA is more practical than ELISA for oxyfluorfen. In addition, the chemiluminescence signal can be measured directly after 10 min of substrate addition, but the ELISA requires 20–30 min of incubation and stop steps. Therefore, CLEIA shortens the overall analysis program and detection time compared with ELISA.

## Conflicts of interest

There are no conflicts to declare.

## Supplementary Material

RA-008-C7RA12445G-s001
