# Supplementary material for: Development of immunoassays for detecting oxyfluorfen residue in agricultural and environmental samples
Source: RSC Adv. 2018 Jan 30;8(9):5020–5. doi: 10.1039/c7ra12445g (PMC9078040; doi:10.1039/c7ra12445g)
Supplement: RA-008-C7RA12445G-s001 [file RA-008-C7RA12445G-s001.pdf]

Electronic Supplementary Material (ESI) for RSC Advances.
This journal is © The Royal Society of Chemistry 2018

Electronic Supplementary Information

Development of immunoassays for detecting oxyfluorfen residue in agricultural and environmental samples

Enze Sheng [a, b], Mei Du [a, b], Jiachuan Yang [a, b], Xiude Hua [a, b], Minghua Wang [a, b,*]

[a]Department of Pesticide Science, College of Plant Protection, Nanjing Agricultural University, Nanjing 210095, P. R. China

[b]State & Local Joint Engineering Research Center of Green Pesticide Invention and Application, Nanjing 210095, P. R. China

[*]Corresponding author. Tel.: +86 25 84395479. Fax: +86 25 84395479. E-mail address: wangmha@njau.edu.cn (M.W.)

Table S1 Effect of methanol content, ionic strength and pH value on immunoassay

Fig. S1 The ESI-MS spectrogram of the hapten of oxyfluorfen

Fig. S2 The ^{1}H-NMR spectrogram of the hapten of oxyfluorfen

Fig. S3 The ultraviolet spectrogram of OVA, BSA and the conjugates with oxyfluorfen hapten

Fig. S4 Matrix effect of samples on the sensitivity of the ELISA.

Fig. S5 Matrix effect of samples on the sensitivity of the CLEIA.

Table S1 Effect of methanol content, ionic strength and pH value on immunoassay

Factors		ELISA			CLEIA		
		A_{max}/IC_{50}	IC_{50} (mg/L)	R^2	RLU_{max}/IC_{50}	IC_{50} (mg/L)	R^2
Methanol (v/v, %)	0	2.091	0.531	0.978	380021	0.153	0.976
	10	2.355	0.482	0.988	518317	0.063	0.985
	20	3.457	0.321	0.976	489924	0.079	0.968
	30	2.738	0.413	0.965	464409	0.083	0.977
	40	1.914	0.594	0.958	320214	0.121	0.983
	50	1.693	0.674	0.963	242680	0.147	0.984
Na^{+}(mol/L)	0.1	2.155	0.382	0.944	537545	0.066	0.981
	0.2	4.146	0.315	0.965	521735	0.068	0.972
	0.3	4.187	0.273	0.982	626087	0.057	0.994
	0.4	9.412	0.127	0.981	690320	0.053	0.985
	0.5	12.43	0.0894	0.987	740191	0.047	0.991
	0.6	7.854	0.142	0.973	695450	0.051	0.978
pH value	4.5	13.3	0.076	0.954	844452	0.042	0.983
	5.5	16.11	0.069	0.968	1108757	0.033	0.982
	6.5	16.58	0.065	0.982	1306678	0.028	0.996
	7.5	12.34	0.073	0.988	1015944	0.036	0.971
	8.5	11.91	0.084	0.967	888097	0.041	0.984
	9.5	8.692	0.13	0.957	812044	0.045	0.963

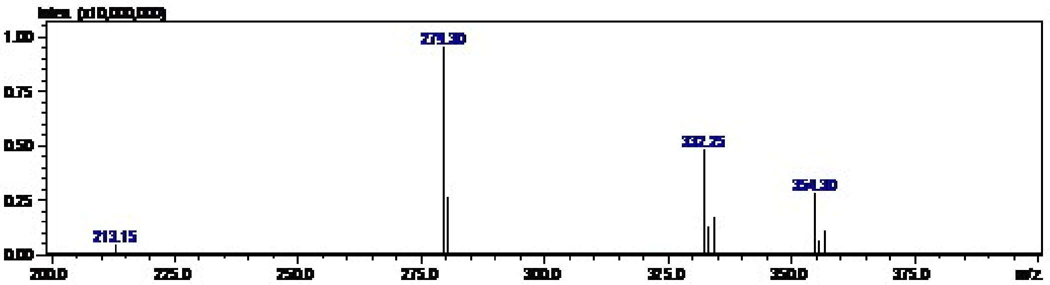

Fig. S1 The ESI-MS spectrogram of the hapten of oxyfluorfen

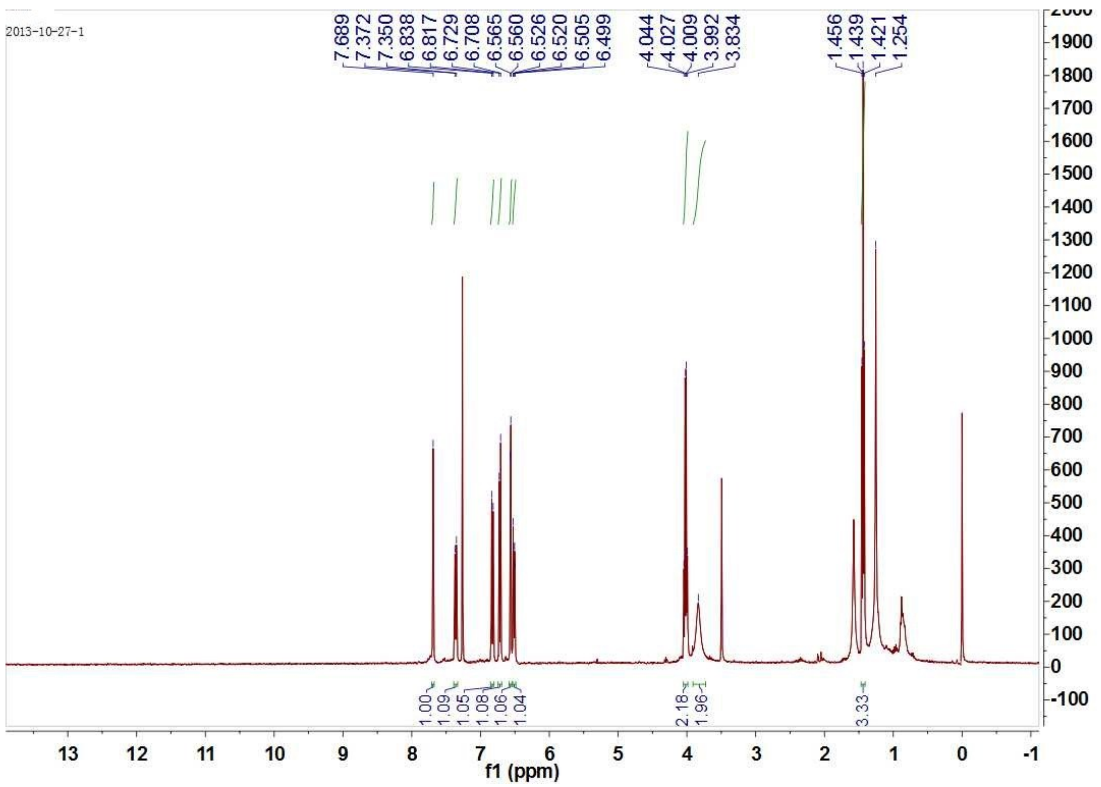

Fig. S2 The ^{1}H-NMR spectrogram of the hapten of oxyfluorfen

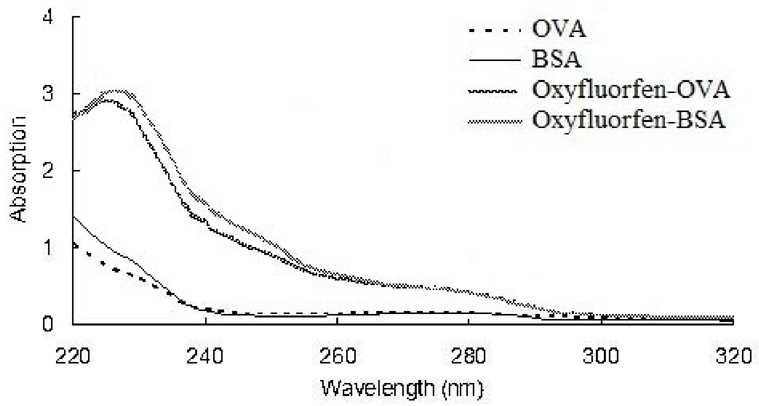

Fig. S3 The ultraviolet spectrogram of OVA, BSA and the conjugates with oxyfluorfen hapten

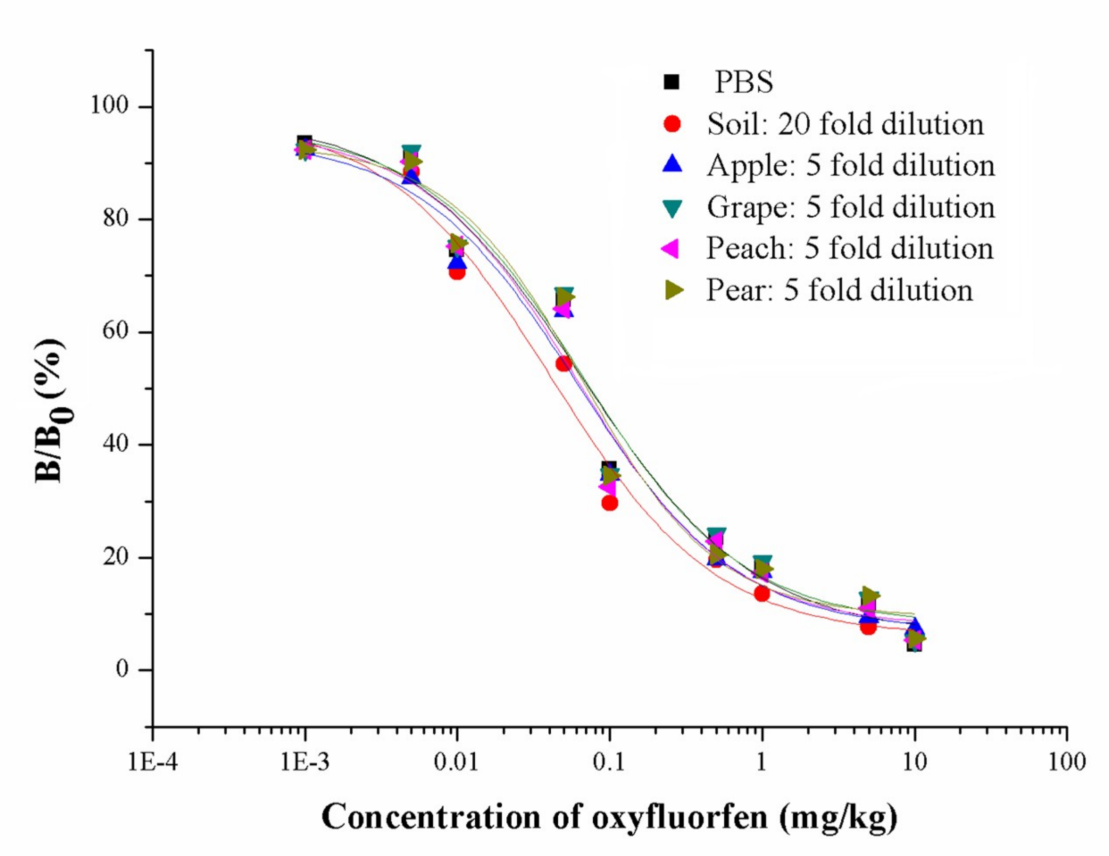

Fig. S4 Matrix effect of samples on the sensitivity of ELISA.

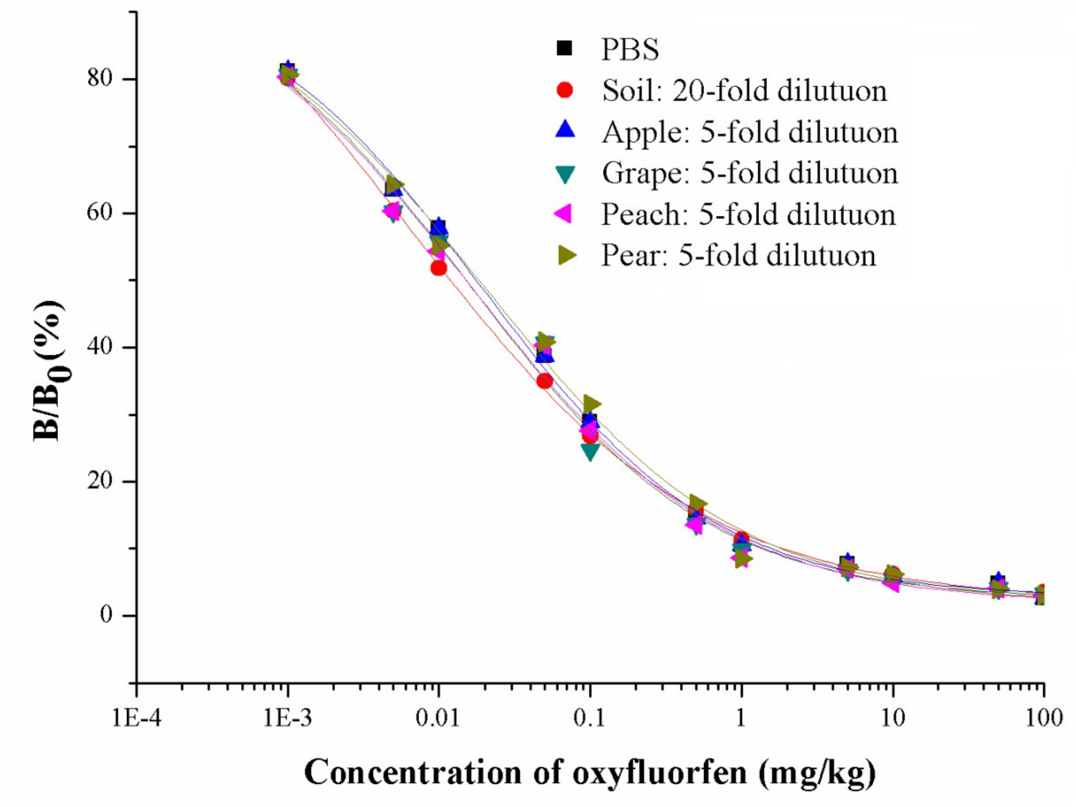

Fig. S5 Matrix effect of samples on the sensitivity of CLEIA.
